# Methylation in the TAC1 Gene Promoter Is Associated with the Transition from Acute Pulmonary Embolism to Chronic Thromboembolic Pulmonary Hypertension

**DOI:** 10.3390/ijms27156913

**Published:** 2026-08-01

**Authors:** Leslie Marisol González-Hermosillo, Guillermo Cueto-Robledo, Javier Gaytan-Cervantes, Dulce Iliana Navarro-Vergara, María Berenice Torres-Rojas, Marisol García-Cesar, Oscar Pérez-Méndez, José Manuel Fragoso, Nallely Bueno-Hernández, Arturo Cérbulo-Vázquez, Galileo Escobedo

**Affiliations:** 1 Programa de Doctorado en Ciencias Biomédicas, Unidad de Posgrado, Universidad Nacional Autónoma de México, Ciudad de México 04510, Mexico; glez.hermosillo.m@gmail.com; 2 Laboratorio de Inmunometabolismo, Dirección de Investigación, Hospital General de México “Dr. Eduardo Liceaga”, Ciudad de México 06720, Mexico; 3 Urgencias Cardiorrespiratorias, Hospital General de México “Dr. Eduardo Liceaga”, Ciudad de México 06720, Mexico; gmocue3@hotmail.com (G.C.-R.); dulceiliana@hotmail.com (D.I.N.-V.); beretorres.m.d@hotmail.com (M.B.T.-R.); shak_mary@hotmail.com (M.G.-C.); 4 Clínica de Circulación Pulmonar, Hospital General de México “Dr. Eduardo Liceaga”, Ciudad de México 06720, Mexico; 5 Facultad de Medicina, Universidad Nacional Autónoma de México, Ciudad de México 04510, Mexico; 6 Laboratorio de Secuenciación, División de Desarrollo de la Investigación, Centro Médico Nacional Siglo XXI, Instituto Mexicano del Seguro Social, Ciudad de México 06720, Mexico; alternativesplicing@gmail.com; 7 Escuela de Ingeniería y Ciencias, Tecnológico de Monterrey, Ciudad de México 14380, Mexico; oscar.perez.m@tec.mx; 8 Departamento de Biología Molecular, Instituto Nacional de Cardiología Ignacio Chávez, Juan Badiano No. 1, Tlalpan, Ciudad de México 14080, Mexico; mfragoso1275@yahoo.com.mx; 9 Laboratorio de Proteómica y Metabolómica, Dirección de Investigación, Hospital General de México “Dr. Eduardo Liceaga”, Ciudad de México 06720, Mexico; nallely_bh5@yahoo.com.mx (N.B.-H.); cerbulo@hotmail.com (A.C.-V.)

**Keywords:** acute pulmonary embolism, chronic thromboembolic pulmonary hypertension, tachykinin precursor 1, methylation, TAC1 gene expression, mixed venous oxygen saturation

## Abstract

Emerging evidence suggests that deoxyribonucleic acid (DNA) methylation may be linked to progression from acute pulmonary embolism (APE) to chronic thromboembolic pulmonary hypertension (CTEPH), especially in genes regulating vascular tone. We investigated the association between tachykinin precursor 1 (TAC1) promoter methylation and the APE-to-CTEPH transition in a 6-month ambispective cohort study of 110 patients with confirmed APE. We recorded clinical, laboratory, and hemodynamic parameters at hospital admission and collected 4 mL of peripheral blood for DNA extraction. We quantified the percentage of TAC1 promoter methylation using bisulfite conversion and methylation-specific polymerase chain reaction (PCR), also assessing TAC1 gene expression by quantitative PCR. During the 6-month follow-up, 7.2% of patients developed CTEPH. Patients who later progressed to CTEPH had a significant 0.4-fold increase in TAC1 promoter methylation compared to those who did not. Increased methylation was associated with a 1.5-fold decrease in TAC1 gene expression in whole-blood leukocytes from CTEPH patients. TAC1 methylation significantly correlated with mixed venous oxygen saturation (SvO2), D-dimer, and B-type natriuretic peptide concentrations. TAC1 promoter hypermethylation is associated with progression from APE to CTEPH and concurs with TAC1 gene repression, probably compromising systemic oxygenation, thrombus resolution, and cardiac strain.

## 1. Introduction

Acute pulmonary embolism (APE) is a life-threatening disease that occurs when a clot obstructs blood circulation in one or more pulmonary arteries [1]. With opportune treatment using anticoagulation therapy and endovascular approaches, APE patients can have a survival rate above 90% [2]. However, up to 12.7% of survivors can develop chronic thromboembolic pulmonary hypertension (CTEPH) within the first six months after hospital discharge [3]. CTEPH is a long-term complication of APE that occurs after thrombi become organized, fibrotic material, leading to persistent obstruction of pulmonary blood flow with a 3-year mortality exceeding 90% [4].

For many years, several authors have considered that progression from APE to CTEPH results from mere arterial mechanical obstruction, which turns into vascular remodeling and pulmonary hypertension [5,6,7]. However, emerging evidence suggests that molecular factors may also act as key contributors to CTEPH development, especially deoxyribonucleic acid (DNA) methylation [8,9]. DNA methylation is an epigenetic mechanism that silences or promotes gene transcription through hypermethylation or hypomethylation, respectively [10]. Several studies suggest that DNA methylation is associated with the development of pathologic features of CTEPH, including pulmonary vascular remodeling and increased pulmonary arterial pressure [11,12]. In this regard, a recent epigenome-wide association study (EWAS) found that the Cathepsin Z promoter is hypermethylated in patients with pulmonary hypertension, a gene with prominent functions in blood vessel thickening [13]. Another study reported that ErbB signaling pathway genes are hypomethylated in patients with increased pulmonary vascular resistance (PVR) and mean pulmonary arterial pressure (mPAP), both key hemodynamic markers of CTEPH [14]. These data support the notion that DNA methylation is involved in CTEPH pathogenesis; however, studies specifically aimed at assessing methylation patterns during progression from APE to CTEPH remain scarce.

The tachykinin precursor 1 (TAC1) gene encodes four bioactive peptides produced by alternative splicing, including substance P (SP), neurokinin A (NKA), neuropeptide K, and neuropeptide g [15,16]. TAC1 peptides are potent regulators of vascular tone and smooth muscle remodeling [17,18]. TAC1 gene expression is significantly altered in hypertensive responses characterized by blood vessel remodeling and elevated pulmonary pressure in rodent models [19,20]. To date, no studies have examined the impact of TAC1 promoter region methylation on CTEPH progression in humans.

Therefore, our main goal was to investigate the association between TAC1 promoter methylation and the APE-to-CTEPH transition in a cohort of patients with confirmed APE followed for six months.

## 2. Results

After completing the 6-month follow-up in the cohort of subjects with confirmed APE, we found that 7.2% of patients developed CTEPH. At hospital admission, there were no significant differences between the CTEPH and non-CTEPH patient groups in age, sex, or body mass index (BMI) (Table 1). Likewise, we did not find differences in biochemical parameters, risk stratification, prevalence of comorbidities, or anticoagulation therapy between patients with APE who developed CTEPH and those who did not progress to CTEPH (Table 1).

**Figure 1:**
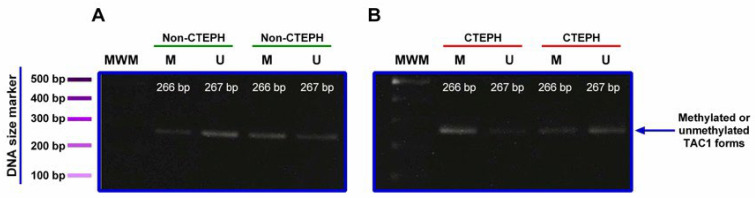
Representative MSP analysis of the TAC1 promoter methylation in whole-blood leukocytes from patients with acute pulmonary embolism who later developed CTEPH and those who did not. (**A**) MSP DNA bands correspond to 2 patients with acute pulmonary embolism who did not develop CTEPH (green lines). (**B**) MSP DNA bands correspond to 2 patients with acute pulmonary embolism who developed CTEPH (red lines). We obtained whole-blood leukocyte samples at hospital admission. We amplified bisulfite-converted genomic DNA using specific primers for both methylated (M) and unmethylated (U) DNA forms, resolved on 2% agarose gels. The blue arrow indicates DNA bands corresponding to methylated or unmethylated forms of the TAC1 gene promoter. Expected product size for M = 266 bp, whereas for U = 267 bp. We illustrate DNA size markers in shades of purple. MWM, molecular weight marker; CTEPH, chronic thromboembolic pulmonary hypertension; M, methylated DNA form; U, unmethylated DNA form.

Figure 1 depicts a representative agarose gel showing methylation-specific PCR (MSP) bands corresponding to the M and U forms of the TAC1 gene promoter, with particular emphasis on comparing patients who developed CTEPH and those who did not. The agarose gel shows that M bands, but not U bands, tend to increase in patients with CTEPH (Figure 1B) compared to non-CTEPH patients (Figure 1A).

**Figure 2:**
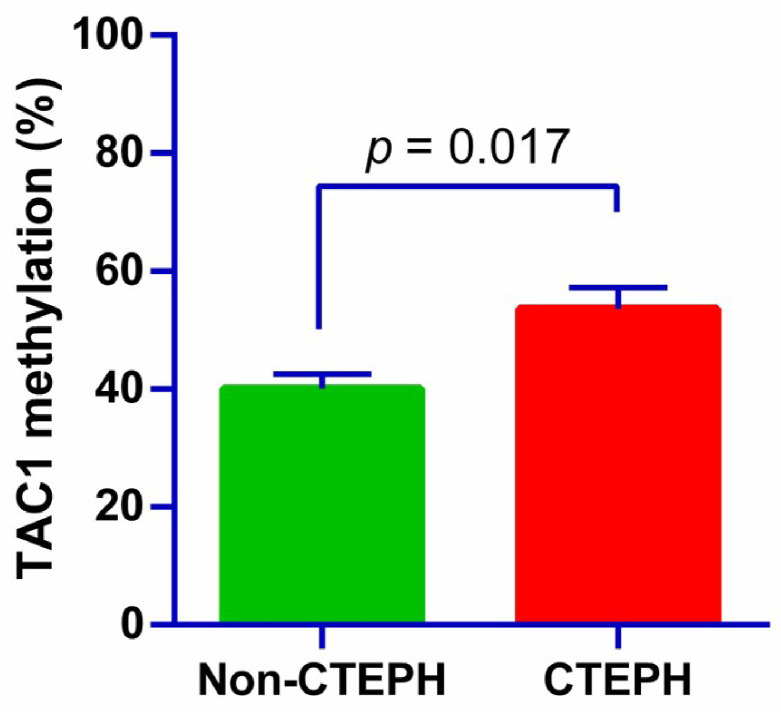
Percentage of TAC1 promoter methylation in whole-blood leukocytes from patients with acute pulmonary embolism who later developed CTEPH and those who did not. Patients who developed CTEPH (*n* = 8) show significantly higher TAC1 gene methylation percentage than patients who did not develop CTEPH (*n* = 102). We obtained whole-blood leukocyte samples at hospital admission. We calculated the TAC1 promoter methylation percentage using the formula: methylation (%) = [FiM/(FiM + FiU)] × 100, where FiM represents the intensity of the methylated product and FiU the intensity of the unmethylated product. We presented data as mean ± standard deviation. We compared data between CTEPH (red bar) and non-CTEPH (green bar) patients using Student’s *t*-test, considering significant differences when *p* < 0.05. Abbreviations: TAC1, tachykinin precursor 1 gene; CTEPH, chronic thromboembolic pulmonary hypertension.

Optical densitometric analysis of M and U DNA bands confirmed that methylation percentage in TAC1 promoter had a significant 0.4-fold increase in patients with APE who developed CTEPH compared to those who did not progress to CTEPH (Figure 2).

Using right heart catheterization (RHC), we also registered hemodynamic variables in all study participants at hospital admission (Table 2). Right ventricle systolic pressure (RVSP), pulmonary artery systolic pressure (PASP), pulmonary artery diastolic pressure (PADP), and mean pulmonary artery pressure (mPAP) were significantly higher in CTEPH patients than in those who did not progress to CTEPH. Notably, the CTEPH patient group showed a significant 2-fold increase in PVR compared to patients with APE who did not develop the complication (Table 2). We found no significant differences in heart rate (HR), right atrium pressure (RAP), pulmonary capillary wedge pressure (PCWP), pulmonary artery compliance (PAC), stroke volume (SV), stroke volume index (SVi), cardiac output (CO), cardiac index (CI), mixed venous partial pressure of oxygen (PvO_2_), mixed venous oxygen saturation (SvO_2_), arterial partial pressure of oxygen (PaO_2_), or arterial oxygen saturation (SaO_2_) between CTEPH and non-CTEPH patients.

Spearman’s rank correlation analysis revealed that SvO_2_ was significantly associated with TAC1 methylation percentage in all participants enrolled in the study with a diagnosis of APE (*ρ* = −0.358, *p* = 0.01) (Table 3). We also observed a significant correlation of TAC1 methylation percentage with D-dimer and BNP serum levels at hospital admission (*ρ* = 0.369, *p* = 0.01, and *ρ* = 0.298, *p* = 0.03, respectively) (Table 3).

**Figure 3:**
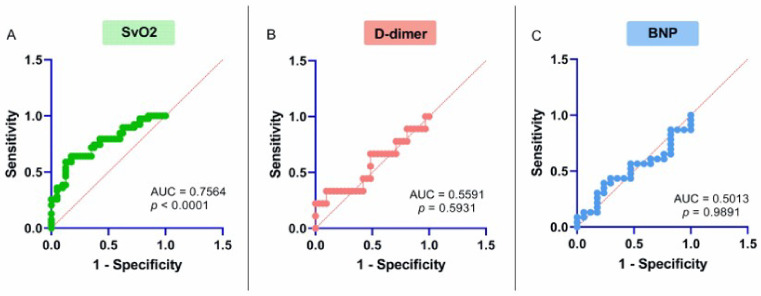
TAC1 promoter methylation percentage differentially discriminates SvO_2_, D-dimer, and BNP in the study subjects at hospital admission. (**A**) TAC1 gene methylation percentage significantly discriminates SvO_2_ with an AUC of 0.75. (**B**) TAC1 gene methylation percentage does not significantly distinguish D-dimer serum levels. (**C**) TAC1 gene methylation percentage does not significantly discriminate BNP serum levels. We generated receiver operating characteristic (ROC) curves to estimate the area under the curve (AUC) and 95% confidence interval (95% CI). We considered significant differences when *p* < 0.05. Abbreviations: SvO_2_, mixed venous oxygen saturation; BNP, B-type natriuretic peptide; AUC, area under the curve.

To expand on these findings beyond mere bivariate correlations, we examined whether TAC1 methylation percentage discriminated SvO_2_, D-dimer, and BNP levels using receiver operating characteristic (ROC) curves. Notably, ROC analysis indicated that TAC1 methylation percentage correlates with abnormally low SvO_2_ levels in the study cohort, with an area under the curve (AUC) of 0.75 (95% confidence interval (CI): 0.65–0.86; *p* < 0.0001) and a statistical power of 0.74 (Figure 3A). Conversely, we found no significant association between TAC1 methylation and high serum levels of D-dimer and BNP in the study population (AUC = 0.55, 95% CI: 0.32–0.79, *p* = 0.59, and AUC = 0.50, 95% CI: 0.31–0.68, *p* = 0.98, respectively) (Figure 3B and 3C, respectively).

**Figure 4:**
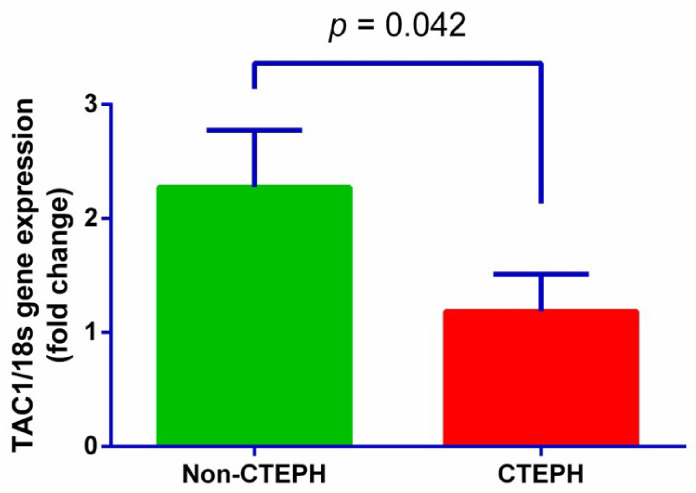
TAC1 gene expression in whole-blood leukocytes from patients with APE or CTEPH. Whole-blood leukocytes from APE patients who later progressed to CTEPH showed a significant 1.5-fold decrease in TAC1 expression compared to whole-blood leukocytes from EPA patients who did not develop CTEPH. The green bar represents non-CTEPH patients, while the red bar represents patients who developed CTEPH. We considered significant differences when *p* < 0.05. Abbreviations: TAC1, tachykinin precursor 1 gene; CTEPH, chronic thromboembolic pulmonary hypertension; APE, acute pulmonary embolism.

After estimating the TAC1 promoter methylation percentage, we confirmed our observations by measuring TAC1 gene expression in whole-blood leukocyte samples from EPA patients who progressed to CTEPH and those who did not. We found that whole-blood leukocytes from patients who progressed to CTEPH showed a significant 1.5-fold decrease in TAC1 expression compared to whole-blood leukocytes from EPA patients who did not develop CTEPH (*p* = 0.042) (Figure 4). These results show that TAC1 gene repression aligns with TAC1 gene promoter hypermethylation in peripheral immune cells of patients with APE-to-CTEPH progression.

Together, our data show a significantly increased methylation percentage in the TAC1 gene promoter in patients with APE who progressed to CTEPH but not in patients with APE who did not develop the complication. Besides concurring with TAC1 gene suppression, TAC1 promoter methylation was correlated with decreased systemic oxygenation, coagulopathy, and heart strain, which could collectively contribute to the transition from APE to CTEPH.

## 3. Discussion

This study provides novel evidence that increased methylation of the TAC1 gene promoter is associated with progression from APE to CTEPH. A few studies have suggested a relationship between gene promoter methylation and the development of CTEPH [21,22]. In this context, a recent study in whole-blood samples from five patients with CTEPH and three healthy controls revealed increased methylation of genes involved in chromatin compaction and decreased methylation of angiogenesis-related genes [8]. Likewise, another study demonstrated that methylation percentage was significantly higher in the hypermethylated in cancer 1 (HIC1) gene in 10 patients with CTEPH compared to 5 controls [14]. Although this information supports a role for promoter methylation in CTEPH, there is not yet solid evidence linking changes in gene methylation patterns to progression from APE to CTEPH. This ambispective study enrolling patients with APE allowed us to collect clinical, biochemical, and hemodynamic parameters, as well as whole-blood DNA samples, at hospital admission. The 6-month follow-up confirmed that patients who later developed CTEPH had a significant increase in TAC1 promoter methylation at the time of hospital admission for APE treatment. We also found that TAC1 gene methylation was associated with low SvO_2_ as well as high D-dimer and BNP levels. This sequence of events suggests that higher TAC1 promoter methylation is associated with the transition from APE to CTEPH, potentially involving systemic oxygenation, coagulopathy, and heart strain.

**Figure 5:**
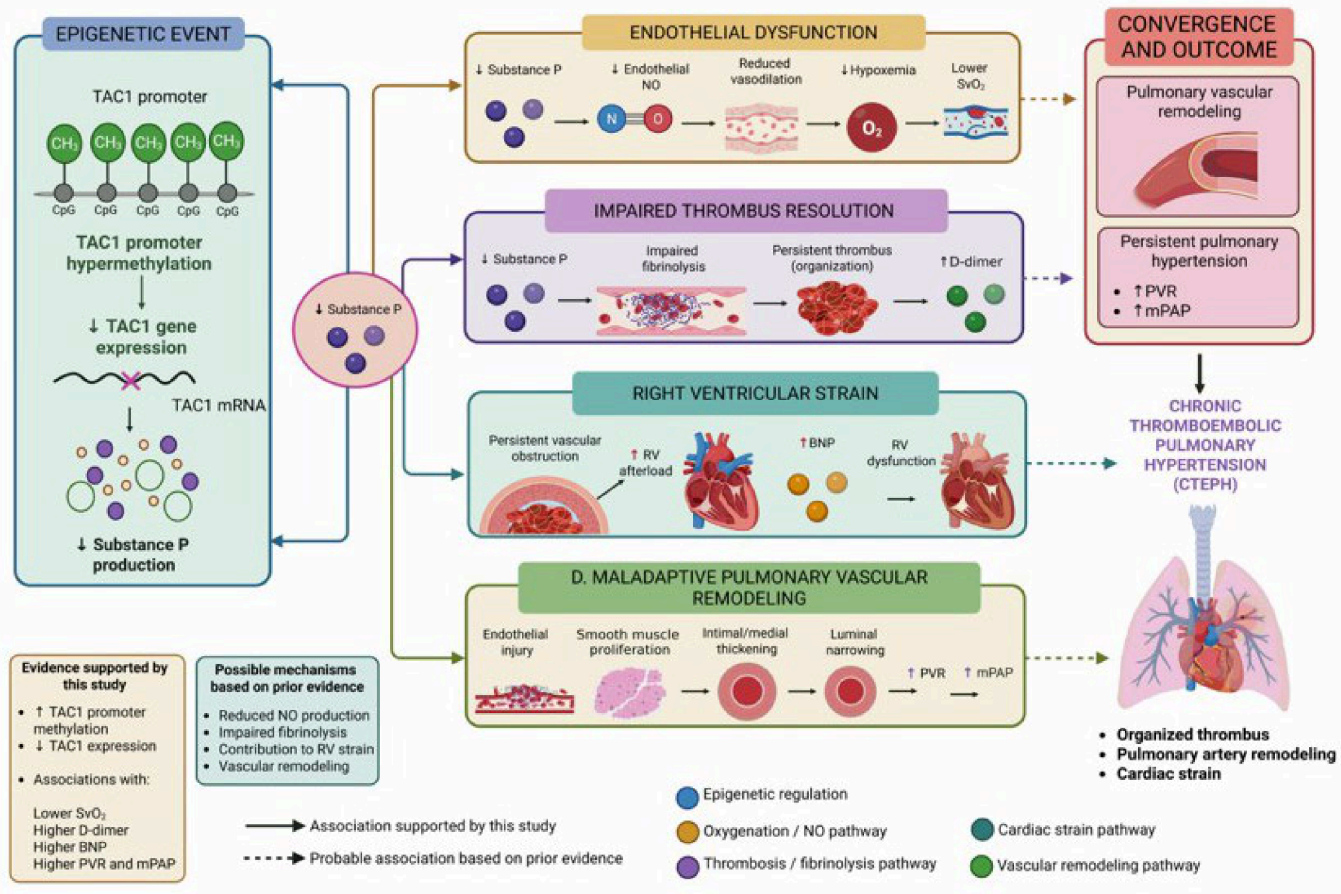
Proposed mechanisms linking TAC1 promoter hypermethylation to the EPA-to-CTEPH transition. Increased methylation in the TAC1 gene promoter (epigenetic regulation marked dark blue) may reduce substance P production, contributing to endothelial dysfunction (oxygenation/NO pathway marked orange), impaired thrombus resolution (thrombosis/fibrinolysis pathway marked purple), right ventricular strain (cardiac strain pathway marked pale blue), or maladaptive pulmonary vascular remodeling (vascular remodeling pathway marked green). These cross-talk interactions may converge into persistent organized thrombi, pulmonary hypertension, pulmonary artery remodeling, cardiac strain, and ultimately CTEPH. Solid lines indicate findings supported by this study, whereas dashed lines represent biologically plausible mechanisms based on prior evidence. Abbreviations: APE, acute pulmonary embolism; CTEPH, chronic thromboembolic pulmonary hypertension; TAC1, tachykinin precursor 1 gene; SvO_2_, mixed venous oxygen saturation; BNP, B-type natriuretic peptide; PVR, pulmonary vascular resistance; mPAP, mean pulmonary artery pressure; NO, nitric oxide; RV, right ventricle. Created with BioRender.com.

SP is one of the most potent tachykinin peptides produced by TAC1 expression [23]. Among its main functions, SP promotes relaxation of vascular smooth muscle cells via endothelial nitric oxide (NO), ultimately leading to arterial vessel dilation [24]. Individuals with a susceptibility to TAC1 promoter hypermethylation may also exhibit decreased TAC1 gene expression, which could compromise SP production, endothelium-derived NO, and the artery’s ability to dilate. This potential scenario concurs with the impaired systemic oxygenation and increased heart injury markers observed in APE patients who further progressed to CTEPH. In parallel, the differential methylation pattern of the TAC1 gene may also be related to thrombus dynamics through SP, either by promoting clot formation or thrombus resolution [25]. The abnormally high D-dimer levels observed during the APE-to-CTEPH transition support this hypothesis, opening new avenues to explore the mechanisms through which TAC1 gene methylation contributes to CTEPH progression. However, although these scenarios are plausible, they still require further experimental confirmation beyond the statistical associations we found in our ambispective cohort study with a 6-month follow-up to confirm APE transition to CTEPH. We summarize these possible mechanisms through which TAC1 promoter hypermethylation may be associated with the APE-to-CTEPH transition in Figure 5.

Along with prior evidence, our findings suggest that progression from APE to CTEPH does not result solely from the body’s inability to resolve thrombus formation [26,27,28]. Rather, the APE-to-CTEPH transition seems to involve an epigenetic predisposition that does not occur equally in all patients but may differentially contribute to endothelial dysfunction, impaired fibrinolysis, heart strain, and maladaptive vascular remodeling [29,30]. In fact, we found that patients with APE who later developed CTEPH exhibited not only significantly higher TAC1 methylation percentages but also increased PVR and mPAP, suggesting a link between TAC1 gene repression and vascular remodeling. In lung arteries, vascular remodeling occurs when blood vessels structurally adapt to sudden changes in blood flow and hemodynamic pressures, leading to endothelial dysfunction and smooth muscle cell proliferation [31,32]. The TAC1 gene also encodes NKA, a neuropeptide that promotes endothelial cell migration, monocyte recruitment, extracellular matrix protein production, and smooth muscle cell expansion via the neurokinin-1 receptor [15,33]. Therefore, TAC1 gene repression by methylation may also be associated with maladaptive vascular remodeling, as evidenced by increased PVR and mPAP in patients with APE who subsequently progressed to CTEPH. These findings support the notion that epigenetic dysregulation may also act as an early molecular determinant of adverse pulmonary vascular remodeling after an acute thromboembolic event [34,35,36]. This concept may help pulmonologists and interventional cardiologists personalize therapeutic strategies to prevent progression to CTEPH, considering the patient’s epigenetic background alongside clinical presentation of APE.

Our results suggest that TAC1 promoter hypermethylation may participate in a pathogenic axis linking systemic hypoperfusion, heart strain, defective thrombus clearance, and pulmonary vascular remodeling, which is associated with APE-to-CTEPH progression. Our findings differ from studies viewing CTEPH as a consequence of a mere mechanical obstruction caused by organized fibrotic thrombi, emphasizing an epigenetic susceptibility component instead [37,38,39]. Considering epigenetics may help understand why only a subset of accurately anticoagulated APE survivors develops CTEPH despite similar embolic burden and conventional risk scores.

DNA methylation is an epigenetic mechanism in which a methyl group is added to the cytosine within the DNA molecule, thereby altering gene expression without altering the gene sequence [40]. Prior cross-sectional studies have shown specific methylation signatures in CTEPH, particularly in genes regulating coagulation, fibrinolysis, inflammation, and vascular remodeling [30,41,42]. However, no cohort studies have prospectively examined whether gene methylation may favor the transition from APE to CTEPH by affecting key pathologic processes such as coagulation, inflammation, or vascular remodeling. To the best of our knowledge, this is the first study specifically designed to enroll patients who survived an episode of APE and follow them prospectively for six months to identify those who subsequently developed CTEPH. This prospective longitudinal design enabled us to characterize the association between TAC1 promoter methylation and progression from APE to CTEPH, providing key information to investigate epigenetic mechanisms underlying disease transition. Interestingly, our findings also showed that TAC1 gene methylation concurs with TAC1 gene suppression in whole-blood leukocytes. This biological consistency across gene methylation and expression supports the notion that TAC1 epigenetic regulation is not only a peripheral biomarker but may also reflect crucial pathological processes involved in the transition to CTEPH. To some extent, these findings suggest that peripheral cell-derived methylation signatures can capture molecular alterations occurring in pulmonary vascular tissues, highlighting their potential value as minimally invasive biomarkers for CTEPH progression. These results open an interesting avenue for exploring the potential utility of gene methylation in predicting the transition from APE to CTEPH, a notion that encourages further research combining molecular and clinical tools to understand CTEPH development.

We must acknowledge several limitations to the study. First, the relatively small number of patients progressing to CTEPH. The fact that only eight participants developed CTEPH during the 6-month follow-up may limit the statistical ability to detect modest effects, even though the observed statistical power suggests robust results. Nevertheless, readers should interpret these results with caution until confirmation in larger multicenter cohorts that allow extrapolation to other tertiary referral hospitals. Second, we assessed TAC1 promoter methylation using peripheral leukocyte DNA. While peripheral leukocyte DNA enables non-invasive assessment of gene methylation, the methylation profile may differ between peripheral leukocytes and vascular tissue. However, it is crucial to note that there is no ethical justification for biopsying pulmonary arteries in patients with APE; thus, access to peripheral leukocyte DNA provides an alternative for studying epigenetic modifications when tissue isolation is limited. Third, although we carefully standardized our semi-quantitative MSP-densitometric approach, this methodology does not provide the single-CpG quantitative resolution achieved by pyrosequencing or methylation-sensitive high-resolution melting (MS-HRM). Therefore, our results should be interpreted with appropriate caution and warrant confirmation in future studies using these quantitative methylation techniques in larger, independent cohorts. Fourth, although the ambispective study design allows us to group patients prospectively while analyzing data retrospectively, our observational findings cannot establish causality or confirm that TAC1 promoter methylation directly causes CTEPH progression. Despite these limitations, our data show that TAC1 promoter methylation is associated with the transition from APE to CTEPH, probably linked to systemic hypoperfusion, heart strain, defective thrombus clearance, and pulmonary vascular remodeling. Future multicenter studies integrating methylation, transcriptomic, and functional peptide analyses are warranted to clarify the role of TAC1 gene methylation in preventing CTEPH development in patients with APE.

## 4. Materials and Methods

### 4.1. Study Design

We conducted a 6-month ambispective, cohort study and enrolled 110 patients with a diagnosis of APE by computed tomography pulmonary angiography (CTPA), according to the European Society of Cardiology (ESC) guidelines [43]. The study participants attended the Department of Cardiorespiratory Urgencies and received thrombolysis, CDT, and standard-of-care anticoagulation with low-molecular-weight heparin (LMWH) for 5 days, followed by rivaroxaban for 3 months. After hospital discharge, we followed up with all enrolled patients for 6 months, including weekly phone calls and monthly medical appointments. If during the first 6-month follow-up, patients presented with dyspnea, functional limitation, or clinical signs of right heart failure, we immediately performed transthoracic echocardiography, a lung ventilation/perfusion (V/Q) scan, and right heart catheterization (RHC) to confirm the diagnosis of CTEPH.

**Figure 6:**
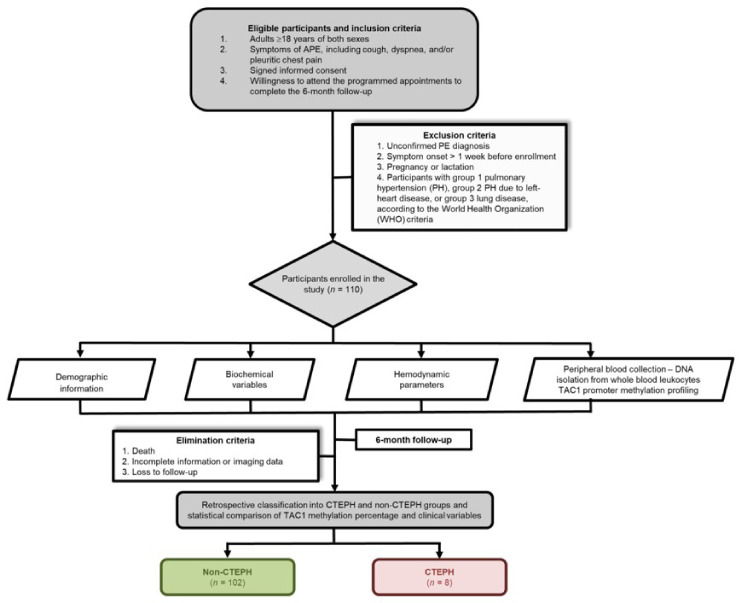
Flowchart illustrating the selection process of the study subjects. At hospital admission, patients with CTPA-confirmed APE were enrolled, and their clinical, laboratory, and hemodynamic parameters were recorded, along with peripheral blood collection for DNA isolation and TAC1 promoter methylation analysis. All enrolled patients were prospectively followed for 6 months through weekly phone calls and monthly medical appointments. Patients presenting dyspnea, functional limitation, or signs of right heart failure underwent transthoracic echocardiography, lung ventilation/perfusion scan, and right heart catheterization to confirm CTEPH. After completing follow-up, clinical, biochemical, hemodynamic, and methylation data were analyzed retrospectively, classifying the cohort into patients who developed CTEPH (*n* = 8) and those who did not (*n* = 102). Abbreviations: CTEPH, chronic thromboembolic pulmonary hypertension; APE, acute pulmonary embolism; DNA, deoxyribonucleic acid.

Diagnostic criteria for CTEPH included mean pulmonary arterial pressure (mPAP) ≥ 20 mmHg, pulmonary capillary wedge pressure (PCWP) ≤ 15 mmHg, persistent perfusion defects on lung V/Q scan, and evidence of chronic pulmonary thromboembolism by CTPA or digital subtraction angiography for at least 3 months, despite effective anticoagulation (Figure 6).

We excluded participants with group 1 pulmonary hypertension (PH), group 2 PH due to left-heart disease, or group 3 lung disease, according to the World Health Organization (WHO) criteria [44]. All enrolled patients agreed to participate in the study by signing the informed consent letter. The Institutional Ethics Committee of the General Hospital of Mexico approved the study protocol under registration number DI/23/503/03/16, supervising the conduct of the study in accordance with the Declaration of Helsinki 1989.

### 4.2. Clinical, Laboratory, and Hemodynamic Parameters

We registered clinical, laboratory, and hemodynamic data using the electronic records of the General Hospital of Mexico. Clinical data included age, sex, body mass index (BMI), intermediate-high/high risk stratification for APE, PESI score, anticoagulation drug therapy, thrombolysis, catheter-directed thrombolytic therapy (CDT), and diagnosis of systemic arterial hypertension, *Diabetes mellitus*, stroke, or dyslipidemia. Laboratory parameters included total leukocyte count, hemoglobin, sodium, troponin I, lactate, D-dimer, B-type natriuretic peptide (BNP), creatinine, uric acid, total bilirubin, total cholesterol, low-density lipoproteins (LDL), high-density lipoproteins (HDL), and triglycerides. Hemodynamic variables included heart rate (HR), right atrium pressure (RAP), right ventricle systolic pressure (RVSP), right ventricle diastolic pressure (RVDP), pulmonary artery systolic pressure (PASP), pulmonary artery diastolic pressure (PADP), mPAP, PCWP, pulmonary artery compliance (PAC), stroke volume (SV), stroke volume index (SVi), cardiac output (CO), cardiac index (CI), pulmonary vascular resistance (PVR), mixed venous oxygen saturation (SvO_2_), arterial oxygen saturation (SaO_2_), mixed venous partial pressure of oxygen (PvO_2_), and arterial partial pressure of oxygen (PaO_2_).

### 4.3. Sample Collection and DNA Isolation

We collected 4 mL of peripheral blood from all enrolled participants at hospital admission. We isolated genomic DNA (gDNA) from the total leukocyte fraction using the Quick-DNA miniprep kit (Zymo Research, Irvine, CA, USA) following the manufacturer’s instructions. We assessed DNA concentration and purity using a NanoDrop 2000 spectrophotometer (Thermo Scientific, Waltham, MA, USA) and calculated the A260/A280 ratio.

### 4.4. Bisulfite Conversion of DNA

To assess the methylation pattern of the TAC1 promoter, we converted 500 ng of gDNA with sodium bisulfite using the EpiJET Bisulfite Conversion Kit (Thermo Scientific, Waltham, MA, USA), according to the manufacturer’s instructions. Bisulfite treatment of gDNA converts unmethylated cytosines to uracil while leaving methylated cytosines unchanged, enabling the differentiation between methylated and unmethylated DNA promoter regions via polymerase chain reaction (PCR).

### 4.5. TAC1 Promoter and Primer Design

We selected the CpG islands located within the promoter region of the human TAC1 gene. We retrieved the TAC1 promoter sequence using the Ensembl Genome Browser and the UCSC Genome Browser with the reference genome GRCh38. We specifically targeted the promoter region 1000 bp upstream of the transcription start site (TSS), where CpG dinucleotide density is highest [45]. We designed two sets of primers for methylation-specific PCR using the MethPrimer software V1.0 [46] to differentiate between methylated and unmethylated templates.

### 4.6. Methylation-Specific PCR (MSP)

We assessed TAC1 methylation status by endpoint methylation-specific PCR (MSP) using primers specific for methylated (M) and unmethylated (U) TAC1 promoter regions. The primer sequences for methylated TAC1 promoter are as follows: Forward 5′-TAGGTGTTATGAAATTTAGTTTCGA-3′ Reverse 5′-TTAAACCTCCAAACCCACGTA-3′. The primer sequences for the unmethylated TAC1 promoter are as follows: Forward 5′-TAGGTGTTATGAAATTTAGTTTTGA-3′ Reverse 5′-CTTAAACTCCAAACC-CACATA-3′. The methylated and unmethylated primers yield amplicons of 266 bp and 267 bp, respectively. We performed MSP using 25 μL of reaction buffer containing 12.5 ng of bisulfite-treated DNA, GoTaq^®^ Green Master Mix (Promega, Madison, WI, USA), and specific primers. The thermal cycling conditions consisted of an initial denaturation step at 95 °C for 2 min, followed by 40 cycles of 95 °C for 30 s, 50 °C for 30 s, and 72 °C for 30 s.

### 4.7. Calculation of DNA Methylation Percentage

We visualized PCR products by electrophoresis on a 2% agarose gel stained with SYBR Safe, with the band in the M lane corresponding to the methylated DNA form and the band in the U lane corresponding to the unmethylated DNA form. We captured methylated and unmethylated DNA bands using a digital gel documentation system and ImageJ software 1.41 for densitometric quantification of band fluorescence intensities (Fi) (NIH, Bethesda, MD, USA). We normalized M- and U-band intensities to the total leukocyte count at hospital admission for each patient. We calculated the TAC1 promoter methylation percentage as follows: methylation (%) = [FiM/(FiM + FiU)] × 100, where FiM represents the intensity of the methylated product and FiU the intensity of the unmethylated product, as previously reported [47]. We tested the accuracy of our MSP semi-quantitative method using available standard types from genomic DNA of HCT116 DKO cells purchased from Zymo Research (Zymo Research Corporation, Irvine, CA, USA).

### 4.8. TAC1 Expression by Quantitative PCR

In addition to methylation percentage in the TAC1 promoter, we measured TAC1 gene expression in whole-blood leukocytes from APE patients who did not develop CTEPH and from those who progressed to CTEPH. Briefly, we placed whole-blood leukocytes in TRIzol Reagent (Thermo Scientific, Waltham, MA, USA) on ice. Then, we isolated total RNA samples using the phenol/chloroform/guanidine isothiocyanate method according to the manufacturer’s instructions (Thermo Scientific, Waltham, MA, USA). After quantifying RNA samples by NanoDrop 2000 spectrophotometer, we synthesized complementary DNA (cDNA) from 500 ng of total RNA using the M-MLV Reverse Transcriptase system with oligo(dT) primers (Thermo Scientific, Waltham, MA, USA). We performed real-time quantitative PCR (qPCR) using cDNA, Fast SYBR^TM^ Green Master Mix (Thermo Scientific, Waltham, MA, USA), and gene-specific primers for TAC1, forward 5′-ACTGTCCGTCGCAAAATCCA-3′; reverse 5′-AGAGCCTTTAACAGGGCCAC-3′. Each 20 µL reaction contained 20 ng of cDNA and 200 nM of each primer. Cycling conditions were 95 °C for 2 min, followed by 40 cycles of 95 °C for 20 s and 61 °C for 30 s. We used 18S ribosomal RNA as the reference gene, primer forward 5′-CGCGGTTCTATTTTGTTGGT-3′ and primer reverse 5′-AGTCGGCATCGTTTATGGTC-3′ and calculated relative TAC1 expression as fold change. Samples were run in duplicate.

### 4.9. Statistics

We assessed the data normality using the Shapiro–Wilk test. We expressed continuous variables as mean ± standard deviation or median ± interquartile range and categorical variables as absolute frequencies or percentages. We compared data from APE patients who did not develop CTEPH with those from APE patients who developed CTEPH using Student’s *t*-test. We analyzed categorical variables using Fisher’s exact test and examined statistical correlations using the Pearson correlation model for calculating coefficients (r) and *p*-values. We performed intragroup comparisons of densitometric band intensities between M and U DNA bands using the Wilcoxon signed-rank test and one-way ANOVA followed by Tukey’s post hoc test for multiple comparisons. We generated receiver operating characteristic (ROC) curves to estimate the area under the curve (AUC) and 95% confidence interval (95% CI), using the Youden index to determine cut-off points, sensitivity, and specificity, and calculating the statistical power using the Hanley and McNeil framework. We used GraphPad Prism version 9.0 (GraphPad Software, San Diego, CA, USA) for statistical analyses, with *p*-values < 0.05 considered significant.

## 5. Conclusions

This study provides evidence that TAC1 promoter hypermethylation is associated with the transition from APE to CTEPH. Increased methylation was also consistent with decreased TAC1 gene expression in whole-blood leukocytes. TAC1 promoter hypermethylation may be involved in the APE-to-CTEPH transition through compromising systemic oxygenation, thrombus resolution, and cardiac strain. Our findings support the notion that epigenetic alterations may contribute to CTEPH pathogenesis beyond mere mechanical thrombotic obstruction, encouraging further multicenter studies to identify APE patients at higher risk of developing CTEPH.

## Figures and Tables

**Table 1:** Demographic, clinical, and biochemical characteristics of the study subjects at hospital admission.

Variable	Total Enrolled Patients with Confirmed APE (*n* = 110)	CTEPH After 6-Month Follow-Up		*p*-Value
		No (*n* = 102)	Yes (*n* = 8)	
Age, years	54.2 ± 16.4	56.2 ± 17.6	53.7 ± 16.4	0.702
Sex (male), *n* (%)	44 (40)	38 (37.2)	4 (50)	0.519
Body mass index, kg/m^2^	31.9 ± 8.6	31.9 ± 7.2	31.7 ± 13.6	0.947
Hemoglobin g/dL	14.3 ± 3.7	13.7 ± 3.8	16.4 ± 2.3	0.068
Sodium mEq/L	137.5 ± 5.1	138.1 ± 5.5	135.4 ± 2.3	0.191
Troponin I ng/mL	202.7 ± 315.8	183.5 ± 300.5	290.4 ± 392.6	0.424
Lactate mmol/L	1.9 ± 1.0	1.8 ± 1.1	2.5 ± 0.5	0.137
Leukocytes/μL	10.3 ± 5.2	10.0 ± 5.6	11.4 ± 3.2	0.537
D-dimer ng/mL	16,433.3 ± 21,374.6	11,849.7 ± 8573.3	33,049.1 ± 40,657.8	**0.011**
BNP pg/mL	686.7 ± 1020.9	470.0 ± 830.6	1584.3 ± 1301.5	**0.008**
Creatinine mg/dL	1.3 ± 1.1	1.2 ± 1.1	1.5 ± 1.2	0.478
Uric Acid mg/dL	7.9 ± 3.2	7.6 ± 3.0	9.6 ± 3.9	0.181
Total Bilirubin mg/dL	1.3 ± 1.3	1.3 ± 1.1	1.7 ± 2.0	0.449
Total Cholesterol mg/dL	140.7 ± 44.9	145.3 ± 45.0	111.2 ± 35.0	0.116
LDL mg/dL	86.9 ± 34.7	89.4 ± 35.6	71.4 ± 26.6	0.290
HDL mg/dL	29.6 ± 13.5	30.4 ± 14.0	25.0 ± 9.8	0.417
Triglycerides mg/dL	170.8 ± 130.1	179.5 ± 137.0	115.2 ± 49.7	0.311
Hypertension, *n* (%)	33 (30)	29 (28.4)	3 (37.5)	0.605
Diabetes mellitus, *n* (%)	14 (12.7)	14 (13.7)	0 (0)	0.232
Stroke, *n* (%)	2 (1.8)	2 (1.9)	0 (0)	0.468
Dyslipidemia, *n* (%)	94 (85.4)	92 (90.1)	5 (62.5)	0.475
Risk stratification (intermediate-high/high), *n* (%)	96 (87.2)	86 (84.3)	8 (100)	0.779
Treatment received (anticoagulation/thrombolysis/CDT), *n* (%)	110 (100)	102 (100)	8 (100)	-

We reported data as mean ± standard deviation, absolute numbers, or percentages as appropriate. We compared data using Student’s *t*-test or Fisher’s exact test, considering significant differences when *p* < 0.05. Bolds indicate significant differences. Abbreviations: CTEPH, chronic thromboembolic pulmonary hypertension; APE, acute pulmonary embolism; BNP, B-type natriuretic peptide; LDL, low-density lipoprotein; HDL, high-density lipoprotein; CDT, catheter-directed thrombolysis.

**Table 2:** Hemodynamic parameters of the study subjects at hospital admission.

Variable	Total Enrolled Patients with Confirmed APE (*n* = 110)	CTEPH After 6-Month Follow-Up		*p*-Value
		No (*n* = 102)	Yes (*n* = 8)	
Heart Rate, bpm	100.5 ± 21.2	103.0 ± 21.6	91.1 ± 17.9	0.161
Right Atrium Pressure, mmHg	7.4 ± 5.3	6.6 ± 5.1	10.0 ± 5.4	0.074
Right Ventricle Systolic Pressure, mmHg	52.7 ± 21.3	48.3 ± 19.5	69.8 ± 20.7	**0.009**
Right Ventricle Diastolic Pressure, mmHg	6.5 ± 4.9	5.8 ± 4.6	9.1 ± 5.5	0.096
Pulmonary Artery Systolic Pressure, mmHg	52.2 ± 20.5	48.5 ± 18.9	68.5 ± 20.9	**0.018**
Pulmonary Artery Diastolic Pressure, mmHg	21.5 ± 9.7	19.9 ± 9.2	28.4 ± 9.8	**0.036**
Mean Pulmonary Artery Pressure, mmHg	34.3 ± 13.4	31.7 ± 12.2	44.5 ± 14.2	**0.015**
Pulmonary Capillary Wedge Pressure, mmHg	7.7 ± 5.0	8.2 ± 5.3	5.6 ± 3.1	0.192
Pulmonary Artery Compliance, mL/mmHg	2.4 ± 2.0	2.6 ± 2.1	1.2 ± 0.5	0.137
Stroke Volume, mL/beat	52.7 ± 20.7	53.5 ± 21.3	48.2 ± 18.2	0.571
Stroke Volume Index, mL/beat/m^2^	27.7 ± 9.9	28.0 ± 10.5	26.2 ± 6.4	0.691
Cardiac Output, L/min	5.0 ± 1.6	5.2 ± 1.6	4.3 ± 1.6	0.187
Cardiac Index, L/min/m^2^	2.5 ± 0.8	2.6 ± 0.8	2.1 ± 0.5	0.117
Pulmonary Vascular Resistance Wood Units	6.2 ± 3.7	5.3 ± 3.2	9.6 ± 3.6	**0.003**
PvO_2_, mmHg	37.6 ± 6.1	38.3 ± 5.5	35.0 ± 8.1	0.174
SvO_2_, %	57.2 ± 13.8	58.3 ± 14.0	53.0 ± 13.0	0.337
PaO_2_, mmHg	57.4 ± 10.9	59.0 ± 11.0	50.5 ± 7.7	0.063
SaO_2_, %	84.9 ± 8.2	85.9 ± 7.7	80.5 ± 9.6	0.123

We reported data as mean ± standard deviation. We compared data using Student’s *t*-test, considering significant differences when *p* < 0.05. Bolds indicate significant differences. Abbreviations: CTEPH, chronic thromboembolic pulmonary hypertension; APE, acute pulmonary embolism; PvO_2_, mixed venous partial pressure of oxygen; SvO_2_, mixed venous oxygen saturation; PaO_2_, arterial partial pressure of oxygen; SaO_2_, arterial oxygen saturation.

**Table 3:** Pearson correlation analysis of TAC1 promoter methylation percentage with hemodynamic and biochemical parameters in the study subjects at hospital admission.

	TAC1 Promoter Methylation Percentage	
	*r*	*p*
Heart Rate, rpm	−0.113	0.244
Right Atrium Pressure, mmHg	−0.062	0.353
Right Ventricle Systolic Pressure, mmHg	0.185	0.128
Right Ventricle Diastolic Pressure, mmHg	−0.021	0.448
Pulmonary Artery Systolic Pressure, mmHg	0.183	0.135
Pulmonary Artery Diastolic Pressure, mmHg	0.054	0.371
Mean Pulmonary Artery Pressure, mmHg	0.115	0.241
Pulmonary Capillary Wedge Pressure, mmHg	0.001	0.496
Pulmonary Artery Compliance, mL/mmHg	−0.025	0.440
Stroke Volume, mL/beat	−0.0329	0.424
Stroke Volume Index, mL/beat/m^2^	0.004	0.490
Cardiac Output, L/min	−0.229	0.089
Cardiac Index, L/min/m^2^	−0.123	0.226
Pulmonary Vascular Resistance Wood Units	0.174	0.144
PvO_2_, mmHg	−0.267	0.050
SvO_2_, %	−0.358	**0.012**
PaO2, mmHg	−0.208	0.105
SaO_2_, %	−0.204	0.109
Hemoglobin, g/dL	0.112	0.244
Sodium, mEq/L	−0.083	0.305
Troponin I, ng/mL	0.055	0.367
Lactate, mmol/L	−0.027	0.435
Leukocytes/mL	−0.065	0.345
D-dimer, ng/mL	0.369	**0.012**
BNP, pg/mL	0.298	**0.038**
Creatinine, mg/dL	0.028	0.430
Uric Acid, mg/dL	0.200	0.114
Total Bilirubin, mg/dL	0.014	0.465
Total Cholesterol, mg/dL	0.149	0.188
LDL, mg/dL	0.206	0.110
HDL, mg/dL	0.274	0.050
Triglycerides, mg/dL	−0.031	0.426

We reported data as correlation coefficients (*r*) and *p*-values, using the Pearson correlation model. We considered significant differences when *p* < 0.05. Bolds indicate significant differences. Abbreviations: TAC1, tachykinin precursor 1 gene; CTEPH, chronic thromboembolic pulmonary hypertension; APE, acute pulmonary embolism; PvO_2_, mixed venous partial pressure of oxygen; SvO_2_, mixed venous oxygen saturation; PaO_2_, arterial partial pressure of oxygen; SaO_2_, arterial oxygen saturation; BNP, B-type natriuretic peptide; LDL, low-density lipoprotein; HDL, high-density lipoprotein.

## Data Availability

Data are available upon request.

## Abbreviations

The following abbreviations are used in this manuscript:

APE	Acute Pulmonary Embolism
BMI	Body Mass Index
CDT	Catheter-Directed Thrombolysis
CpG	Cytosine-phosphate-Guanine
CTEPH	Chronic Thromboembolic Pulmonary Hypertension
CTPA	Computed Tomography Pulmonary Angiography
DNA	Deoxyribonucleic Acid
ESC	European Society of Cardiology
gDNA	Genomic DNA
IQR	Interquartile Range
M	Methylated
mPAP	Mean Pulmonary Arterial Pressure
MSP	Methylation-Specific PCR
PAH	Pulmonary Arterial Hypertension
PCR	Polymerase Chain Reaction
PCWP	Pulmonary Capillary Wedge Pressure
PESI	Pulmonary Embolism Severity Index
PVR	Pulmonary Vascular Resistance
RHC	Right Heart Catheterization
SD	Standard Deviation
TAC1	Tachykinin 1
TSS	Transcription Start Site
U	Unmethylated
V/Q	Ventilation/Perfusion
